# Uterine adenosarcoma metastasizing to the retroperitoneum. The impact of vascular involvement

**Published:** 2012-06-18

**Authors:** RI Lazar, T Straja, B Bratucu

**Affiliations:** * Department of Restorative Dentistry, “Carol Davila” University of Medicine and Pharmacy, Bucharest; ** Department of Endodontics, “Carol Davila” University of Medicine and Pharmacy, Bucharest

**Keywords:** retroperitoneal metastasis, mullerian adenosarcoma, vascular involvement, prognostic

## Abstract

**Rationale:** There is little knowledge regarding mullerian adenosarcoma and its metastasizing pattern.

**Aim:** Our objective was to evaluate the impact on the prognostic of the patient brought by tumor metastasizing to the retroperitoneum, to analyze the particularities of the treatment in such cases and to bring a significant change in the early therapeutic attitude to mullerian adenosarcomas.

**Methods and results:** We present a first case report of a hypervascularized retroperitoneal metastasis from an initial low-grade uterine adenosarcoma. The presence of such a metastasis brought the worst prognostic factor to the patient.

**Conclusions:** We consider that in front of a macroscopic polypoid mass there should be an active change in the diagnosis and in the therapeutic attitude. The frequent confusion between similar histopathological entities with different aggressiveness states and specific treatment responses, the poor outcome at advanced tumor stages, frequently in young patients, should trigger a universal remodeling in approaching these tumors.

## Introduction

Among the rare uterine sarcomas (2-9% of all uterine cancers), mullerian adenosarcomas are even less observed and diagnosed, representing only 8% of all uterine mesenchymal malignancies [**1**]. Their frequent confusion with recurrent cervical polyps or with other similar histopathological entities makes their diagnostication even more difficult, impeding an adequate precocious treatment. Mullerian adenosarcomas have been first described by Clement and Scully in 1974, but, due to their rarity, they still represent an elusive aspect of our knowledge. There is still a limited, small number of reports concerning these tumors, randomized trials have not been possible and screening methods are absent [**2**]. Typical mullerian adenosarcomas display a mixed composition: a benign neoplastic glandular component, with a well-differentiated epithelial lining, and a low-grade, malignant sarcomatous stromal structure. Usually, uterine adenosarcomas arise in the endometrium and more seldom within the myometrium. Rare cases of mullerian adenosarcoma of the cervix (4 reports in literature), vagina, ovaries, salpynx, or even more unusual extragenital primary mullerian tumors (peritoneum, abdominal, Douglas pouch, retroperitoneum) have been described [**3-5**]. The majority of the uterine adenosarcomas are confined to the endometrium, but in 15% of cases, they can invade the inner half of the myometrium. The recurrences (in 20-30% of cases) are limited to the vagina, pelvis or abdomen and can appear after a long period of time. The treatment consists of a total hysterectomy with bilateral oophorectomy, but a more conservative attitude is followed in young women. There is no consensus regarding the complementary treatments [**6**]. Here, we present a very rare case of an aggressive retroperitoneal metastasis from a low-grade typical uterine mullerian adenosarcoma. To the best of our knowledge, there are no such reports in literature, the metastasizing rate being very low. Having this case in view, we consider that the current diagnosis and treatment of uterine tumors should be remodeled.

## Case Report

A 78–year-old female patient was admitted to our Surgical Clinic complaining of slow intestinal transit, meteorism, urinary incontinence. Three years before, the patient had a total hysterectomy with bilateral oophorectomy for uterine body mullerian adenosarcoma with invasion into the myometrium and unilateral ovarian metastasis, neglecting further treatment and regular follow-ups. Two years after the initial operation, the patient developed a friable, bleeding vaginal stump recurrence, responsive to radiotherapy (teleradiotherapy-40 Gy and 20 Gy local applications), the imaging data showing a 54/48 (mm) tumor, posterior to the urinary bladder. 

**Fig. 1:**
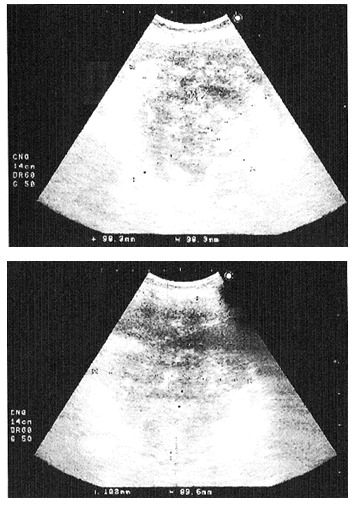
Ultrasound aspects of the retroperitoneal metastasis root of tooth 36

At the admission to our clinic the patient was underweight, with pale teguments, had abdominal meteorism and a palpable, moderately painful, posteriorly fixed tumor, located in the left hypochondrium, with a diameter of approximately 80 (mm). Laboratory tests: moderate anemia. Ultrasound examination (**Fig. 1**): a 140/120 (mm) voluminous, irregular tumor, with Doppler signal, of retroperitoneal and mesenteric topography, with extension to the spleen hilum and to the kidney inferior pole, dislocating the spleen anteriorly and compressing the kidney, with a second degree hydronephrosis.

**Fig. 2:**
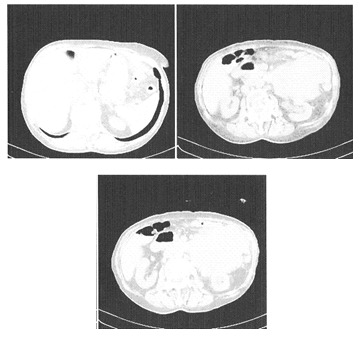
CT aspects of the sarcomatous retroperitoneal metastasis – relationship to the spleen, pancreas, kidney

**Fig. 3:**
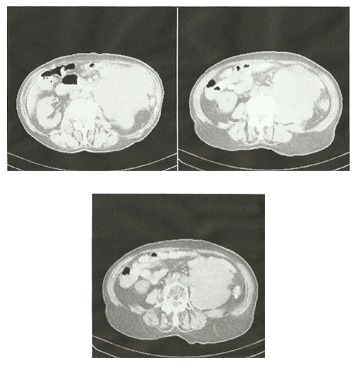
Tumor invasion into the left kidney, ureter and psoas muscle and the relationship between the voluminous retroperitoneal metastasis and the large retroperitoneal vessels.

CT with contrast media (**Fig. 2,3**): a voluminous tumor located in the left flank, of 119/112 (mm), inhomogeneous natively and after contrast media captation, of irregular contour, infiltrative into the surrounding fat tissues, craniocaudally situated from the T12-L1 to the L5-T1 vertebral disk, anterior to the pancreas, compressing the small bowel, invading into the left kidney, left lumbar ureter and into the inferior aspect of left psoas muscle; para-aortic 12 (mm) adenopathy. 

Intraoperatively, a voluminous retroperitoneal tumor was discovered, attached to the base of the mesentery and the first jejunal loop, invading the left kidney, the ureter and the large retroperitoneal blood vessels. The case was considered to be beyond the surgical resources, and the intervention was limited to tumor biopsy. One of the intraoperative complications was represented by an important hemorrhage during tumor mobilization and biopsy. The result of the histopathological examination: sarcomatous metastasis. Postoperatively, after a period of severe anemia and dyselectrolytemia, the patient had a favorable evolution and was released from the hospital. The same year, the patient died due to the progression of the retroperitoneal metastasis.

## Discussions

Usually, mullerian adenosarcomas occur after menopause (median age of 58 years), being described from the second to the tenth decade [**1**], and present as clearly-defined polypoid masses in the uterine cavity. The most frequent symptom is abnormal vaginal bleeding, but also vaginal discharge, pelvic pain, abdominal mass. Risk factors for this tumor are: previous pelvic irradiation, tamoxifen or toremifene treatment for breast cancer, hyperestrogenic state, long term use of contraceptives [**4,7-9**], but other factors escaping our understanding due to the low number of reports may be involved. Typical mullerian adenosarcomas are low malignant tumors, with potential to local recurrence and rare metastasizing consisting of pure sarcoma [**1**]. Their long-term prognostic is not well known due to their extreme rarity. Negative prognostic factors are the degree of myometrium invasion, sarcomatous overgrowth, disease recurrence, presence of heterologous elements [**4-10**]. Preoperative diagnosis of these tumors is rare. Often, these tumors are mistaken for recurrent polyps for long periods of time and their correct diagnosis is severely delayed. Sometimes, the distinction of the uterine tumor type is made on the basis of a curetted material analysis, but the entire tumor may present different benign and malignant features throughout its volume, resulting in a misleading diagnosis. Many similar benign and malignant histopathological entities resemble adenosarcoma (adenofibroma, carcinosarcoma, benign polyps, atypical polypoid adenomyoma, endometrial stromal sarcoma, embryonal rhabdomyosarcoma) and therefore, for a correct therapeutic approach the diagnosis of a uterine polypoid mass should be based on the analysis of the entire hysterectomy piece [**10**]. At low power magnification, adenosarcoma exhibits a leaf-like pattern, resembling phyllodes tumor. The glandular epithelium is active, like a proliferative endometrium, with mitotic activity, despite the advanced age of the patients, and with focal atypia; the stroma is composed of spindled cells arranged in whorls and/or loosely dispersed round cells. Stromal cells form a periglandular cuffing, a zone of maximum mitotic activity and nuclear atypia. Routinely, the diagnosis is made without a stromal cell mitotic count (that in adenosarcoma is more than 1-2 per 10 HPFs), immunohistochemical determinations (ER, PR, CD10, WT1, p53) or DNA aneuploidia/diploidia determination. The features of an aggressive variant, the presence of sarcomatous overgrowth would change the specificity of the medical treatment and follow-up. We consider that in the case of uterine sarcoma, these determinations should be done, together with an analysis of the entire surgical resection piece and the abandon of a fast diagnosis in front of an apparent benign polyp. In the case presented by us, the discovery of a simultaneous ovarian metastasis at the time of the initial operation by histopathological examination, without a macroscopically altered aspect, suggests that maybe, the attitude of conserving the ovaries in younger women is not of the safest and should be, maybe, reconsidered. In this case, the initial tumor presentation displayed many of the characteristic features (age, polypoid aspect, histopathological description, local recurrence after two years), with some factors suggesting a poor prognostic: concomitant metastasis, treatment and follow-up negligence, but also some encouraging: lack of sarcomatous overgrowth, a limited myometrium invasion, lack of risk factors. There still remains a question about the spectacular disappearance of the local recurrence after radiotherapy. But, even more unusual is the metastasizing of the tumor to the retroperitoneum that brought the worst possible prognosis to the patient. Usually, retroperitoneal tumors exhibit a silent growth until they reach gigantic dimensions, with multivisceral and large vessel involvement. Although their treatment is dependent on a radical, even very aggressive resection, this is frequently impossible due to the lack of accessibility into this anatomic space and the difficulty of vascular interventions, the fear of the general surgeon of hypervascularized, almost spontaneously bleeding tumors, very close to or even invading large vessels [**11-13**].. In the presented case, the massive involvement of the abdominal aorta and inferior vena cava were the decisive factor to limit the intervention, although preoperative CT data were inconclusive regarding this aspect. In the territory of retroperitoneal tumors, both primitive and metastases, a multidisciplinary approach is essential: a mixed surgical team, with the presence of a vascular surgeon and appropriate materials for complex vascular interventions, urologists, experts in digestive surgery, but also of radio- and chemo- therapists to apply complementary treatments in selected cases. Not only in retroperitoneal tumors, but also in uterine sarcomas, vascular involvement has been found to be one of the most significant prognostic factors in patient survival [**14**]. A new staging system has been already considered for uterine sarcomas, stressing on their particular biological behavior [**4**]. Having this case in mind, the reports of other authors, and the dependence of the treatment on radical surgery, we propose reconsideration in the current approach of uterine tumors. There should be an increase in the level of susceptibility at the presentation of a patient with a polypoid mass, a limitation, as much as possible, of conservative surgery in younger patients, a mandatory extensive histopathological analysis of the entire hysterectomy specimen with routine stromal mitotic count and immunohistochemical determinations, an obligativity of staging lymphadenectomy during the primary surgery (subject of debate until now), a thorough preoperative and intraoperative vascular involvement assessment, a long period of follow-up after the operation, with an attentive screening of the retroperitoneum too and with a careful imagistic vascular observation. This approach might increase, in time, the diagnosis of these tumor variants that probably occur more frequently than reported and would certainly improve the survival of these, often young patients, before reaching, very fast, a very advanced stage.

**Research Support: **This study has been done in the framework of a FEST project. 
